# Glycosides, Depression and Suicidal Behaviour: The Role of Glycoside-Linked Proteins

**DOI:** 10.3390/molecules16032688

**Published:** 2011-03-23

**Authors:** Gianluca Serafini, Maurizio Pompili, Marco Innamorati, Gloria Giordano, Roberto Tatarelli, David Lester, Paolo Girardi, Yogesh Dwivedi

**Affiliations:** 1 Department of Neuroscience, Mental Health and Sensory Functions, “Sapienza” University of Rome, Suicide Prevention Center, Sant’Andrea Hospital, Via Grottarossa 1035-1039, 00189 Rome, Italy; 2 Villa Rosa, Scientific Medical Research Center, Suore Hospitaliere of the Sacred Heart of Jesus, 01100 Viterbo, Italy; 3 McLean Hospital, Harvard Medical School, Boston, MA 02478, USA; 4 The Richard Stockton College of New Jersey, Jimmie Leeds Road, Pomona, NJ 08240, USA.; 5 Department of Psychiatry, Psychiatric Institute, College of Medicine, University of Illinois at Chicago, Chicago, IL 60612, USA

**Keywords:** glycosides, depression, suicidal behaviour, synaptic plasticity

## Abstract

Nowadays depression and suicide are two of the most important worldwide public health problems. Although their specific molecular mechanisms are still largely unknown, glycosides can play a fundamental role in their pathogenesis. These molecules act presumably through the up-regulation of plasticity-related proteins: probably they can have a presynaptic facilitatory effect, through the activation of several intracellular signaling pathways that include molecules like protein kinase A, Rap-1, cAMP, cADPR and G proteins. These proteins take part in a myriad of brain functions such as cell survival and synaptic plasticity. In depressed suicide victims, it has been found that their activity is strongly decreased, primarily in hippocampus and prefrontal cortex. These studies suggest that glycosides can regulate neuroprotection through Rap-1 and other molecules, and may play a crucial role in the pathophysiology of depression and suicide.

## 1. Introduction

According to the World Health Organization [1], major depression disorder is one of the most widespread diseases in the World, potentially affecting approximately 17% of population lifetime and it still is one of the main leading causes of disability [1,2]. Suicide is also one of the most critical worldwide public health problems, and it accounts for about one million of deaths annually, with devastating socioeconomic costs and consequences [3]. It results from various factors, including psychiatric, biological and environmental factors. There is evidence that suicide rates among mood disorder patients are more than 20-fold higher than in the general population [4].

Although deeply investigated, the precise molecular mechanisms of depression and suicide are still poorly understood, although in recent years significant relevant scientific progress has been made [5,6,7,8,9,10,11]. Interestingly, recent research on the biological perspectives of suicidal behaviour appears to provide a promising approach in identifying reliable biological risk factors.

Many biological theories have postulated that abnormalities in some glycosides and glycoside-linked signal transduction proteins could play a relevant role in the pathophisiology of depression and suicidal behaviour, presumably by mediating a reduced activation and/or expression of specific catalytic and regulatory subunit isoforms of some plasticity-related proteins [12,13,14,15,16,17,18,19,20,21,22,23]. 

In chemistry, glycosides are defined as compounds containing a carbohydrate and a noncarbohydrate residue in the same molecule; specifically, these compounds include a sugar molecule tied up with another chemical at the anomeric carbon via a glycosidic bond [24]. The most common group of glycosides in Nature presumably includes the glycoproteins; in many of the enzyme and glycoproteins, sugars are linked to a protein by O-glycosidic bonds. 

Usually glycosides and glycoproteins play relevant roles in living organisms and represent crucial molecules at the intracellular level. Besides, they also are used as medications. Glycosides may be activated by certain biological enzymes through hydrolysis, resulting in the separation of the sugar portion. When activated, these molecules can act on different intracellular targets (glycoside-linked signal transduction proteins). Glycosides may be classified in different ways or according to different categories but we considered relevant to the present review only some of these molecules, indicated in bold in Table 1. Additionally, we have focused on specific glycoside-linked signal transduction proteins appearing crucial in intracellular signalling in psychiatric illnesses, particularly, in depression and suicide.

Figure 2 summarizes how some intracellular nucleotides may activate Rap-1, a small guanine nucleotide triphosphate (GTP) – binding protein, belonging to the Ras proteins family. Rap-1 is one of the most important substrate of protein kinase A (PKA), a fundamental enzyme in the cyclic adenosine monophosphate (cAMP) signaling pathway.

Hypothesizing that some glycosides and glycoside-linked signal transduction proteins may exert specific intracellular mechanisms and neuroprotective influence on neuronal survival and synaptic plasticity, our aim was to analyze the role of these molecules in depression and suicidal behaviour.

**Table 1:** Nucleobases, nucleosides, nucleotides and nucleic acids.

**Nucleobases**	Purine (Adenine, Guanine, Purine analogue), Pyrimidine (Uracil, Thymine, Cytosine, Pyrimidine analogue)
Ribonucleosides*	Adenosine, Guanosine, Uridine, Cytidine
Deoxyribonucleosides**	Deoxyadenosine, Deoxyguanosine, Thymidine, Deoxyuridine, Deoxycytidine
**Ribonucleotides†**	Monophosphates (**AMP, GMP,** UMP, CMP), diphosphates (**ADP, GDP,** UDP,CDP), triphosphates (**ATP, GTP,** UTP, CTP)
**Deoxyribonucleotides††**	Monophosphates (**dAMP, dGMP,** dUMP, TMP, dCMP), diphosphates (**dADP, dGDP,** TDP, dCDP), triphosphates (**dATP, dGTP,** dTTP, dCTP)
**Cyclic∫**	**cAMP, cGMP,** cGMP, cADPR
**Deoxyribonucleic acids∫∫**	cDNA, cpDNA, gDNA, msDNA, mtDNA
**Ribonucleic acids⌠**	Translation: mRNA, tRNA, rRNA, tmRNA Regulatory: miRNA, RNAi, siRNA, piRNA, RNA processing: snRNA, snoRNA

*A ribonucleoside is a type of nucleoside including ribose as a component. D-Ribose is an aldopentose, a monosaccharide having five carbon atoms with an aldehyde functional group at position 1; **A deoxyribonucleoside is a type of nucleoside including deoxyribose (2-deoxyribose) as a component. It is characterized by the replacement of a hydroxyl group (-OH) by hydrogen (-H) at position 2 of its ribose sugar moiety. Deoxyribose differs from ribose due to the presence of a proton on the 2' carbon rather than an -OH group; † A Ribonucleotide is a nucleotide in which a purine or pyrimidine base is linked to a ribose molecule. Ribonucleotides may have one, two, or three phosphate groups attached to the ribose sugar; †† A deoxyribonucleotide is a single unit of DNA, or deoxyribonucleic acid including three parts: a nitrogenous base, a deoxyribose sugar, and one or more phosphate groups. The nitrogenous base is often bonded to the 1' carbon of the deoxyribose. The phosphate groups bind to the 5' carbon of the sugar; ∫ A cyclic nucleotide is a nucleotide having the phosphate group bonded to two of the sugar's hydroxyl groups resulting in a cyclical or ring structure; ∫∫ cDNA (Complementary DNA), cpDNA (Chloroplasts), gDNA (Genomic deoxyribonucleic acid is chromosomal DNA), msDNA (Multicopy single-stranded DNA), mtDNA (Mitochondrial DNA); ⌠Biologically active RNAs carries information from DNA to the ribosome, the sites of protein synthesis (translation): mRNA (Messenger RNA), tRNA (Transfer RNA), rRNA (Ribosomal RNA), tmRNA (Transfer-messenger RNA). Regulatory RNAs can downregulate gene expression: miRNA, (MicroRNAs), RNAi (RNA interference), siRNA (small interfering RNAs), piRNA (Piwi-interacting RNAs). RNA processing are involved in modifying other RNAs: snRNA (small nuclear RNAs), snoRNA (small nucleolar RNAs).

## 2. Methods

In order to provide a new and timely critical review about glycosides, depression, and suicidal behaviour, we performed a careful MedLine, Excerpta Medica, PsycLit and PsycInfo and Index Medicus search to identify all papers and book chapters in English language during the period between 1980 to 2010. 

We included in the present review only those articles published in peer-reviewed journals, adding an original contribution to the literature. Where a title or an abstract seem to describe a study eligible for inclusion, the full article was obtained and examined to assess its relevance based on the inclusion criteria which were subsequently specified. 

Any discrepancies between the two reviewers who, blind to each other, examined the studies for the possible inclusion were resolved by consultations with a senior author. The search used the following terms: “glycosides”, “nucleosides”, “nucleotides”, “AMP”, “GMP”, “ADP”, “GDP”, “ATP”, “GTP”, “dAMP”, “dGMP”, “c-AMP”, “c-GMP” AND “plasticity-related proteins”, “protein kinase”, “Rap-1”, “cADPR”, “G-proteins”AND “suicide” OR “suicidal behviour” OR “suicidality” AND “depression” OR “mood disorders” OR “Affective disorders” AND “synaptic plasticity”, “cell survival”, “neurogenesis”. 

**Figure 1:**
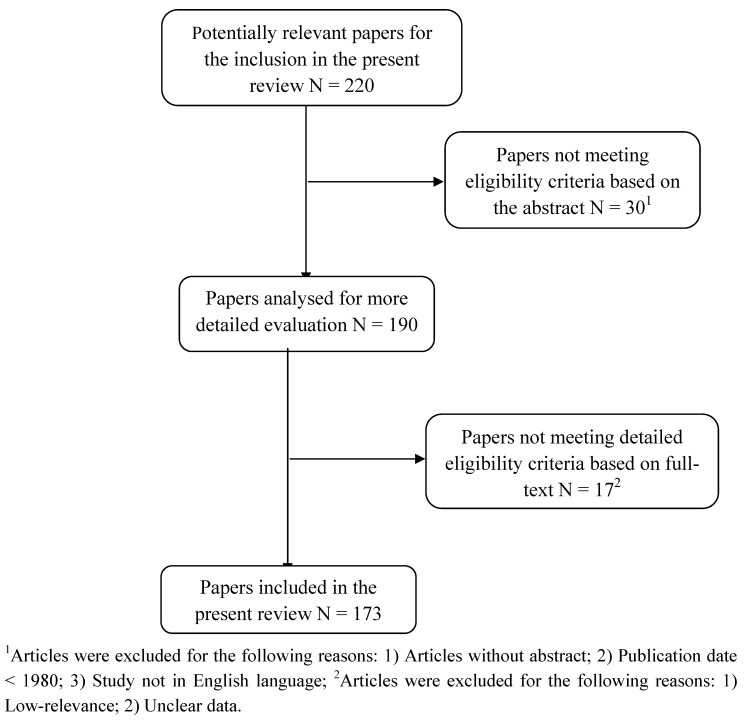
The search strategy for inclusion of studies in the current review.

The combined search strategies yielded a total of 220 abstracts, of which after a complete analysis 190 full-text articles were reviewed and 30 exluded (because they lacked abstracts, or did not explicitly mention suicide and depression). Of 190 which were selected, approximately 173 studies met our inclusion criteria and were included in the present review, the remaining 17 studies having a low-relevance to the main theme, focusing on animal models, or with unclear data regarding materials and methods or number of patients analyzed. Figure 1 summarizes the search strategy used for the inclusion of the main studies considered in the current review.

## 3. G Proteins and Adenylyl Cyclase-cAMP and Their Role in Depression and Suicide

Glycosides may play a central role in different biological functions, presumably by regulating some specific plasticity-related proteins such as G proteins, which may be crucial in the transduction of intracellular signals [25,26,27,28,29] mediated by neurotransmitters, hormones, and neuromodulators receptors. The α subunit binds to guanosine triphosphate (GTP) giving to G proteins the role of receptor effector. If the guanosine diphosphate (GDP) is bound to an α subunit, the G protein is inactive. Receptor-mediated reactions determine the release of GDP from the α subunit and the dissociation of the α subunit from the βγ subunits. These subunits may activate different biological effectors and cause several cellular responses. 

**Figure 2:**
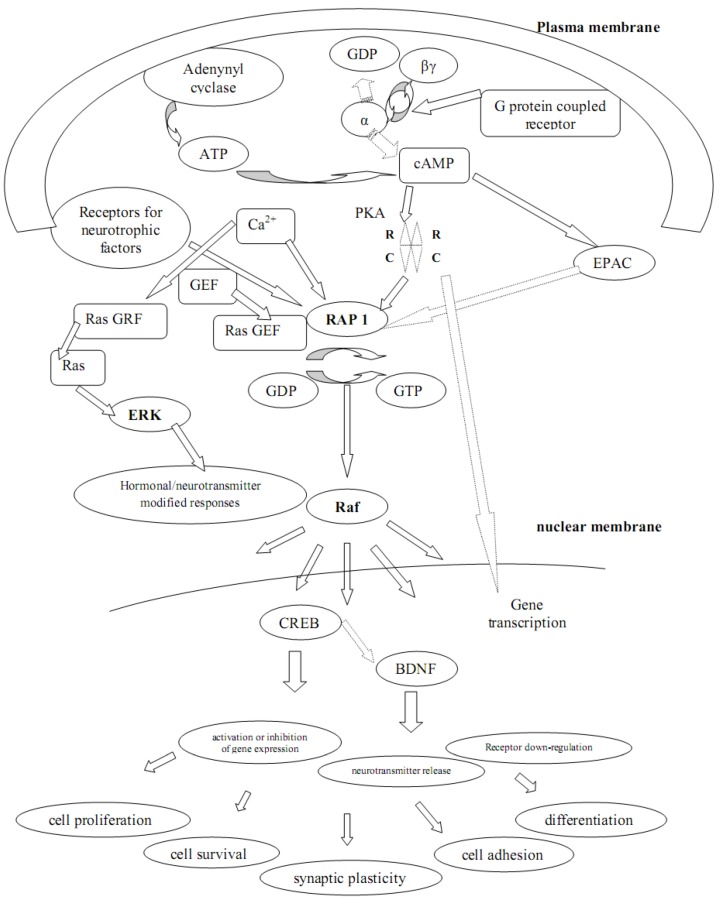
The role of Rap1 in in the transduction of intracellular signals.

Gsα isoform stimulates adenylyl cyclise and induces the conversion of adenosine triphosphate to cyclic AMP (cAMP) which serves as a second messenger and activates the phosphorylation of protein kinase A (PKA). The activation of PKA determines the phosphorylation of different intracellular proteins which modify hormonal or neurotransmitter responses, induces receptor down-regulation, neurotransmitter release, and causes activation or inhibition of gene expression [30,31] (for more details, see Figure 2). 

Some studies have tried to examine the role of the G protein subunits or G protein-mediated functional responses in peripheral tissues and human post-mortem brain of subjects with affective disorders and suicidal behaviour. Basal GTPγ-stimulated or forskolin-stimulated adenylyl cyclase activity had been reported to be reduced in the cerebral cortex of patients with depression [32]. Similarly, the photoaffinity GTP labeling of Gi or Gα was found to be increased in the temporal and parietal cortical areas of patients with depression [33]. Avissar *et al*. [34] found that hypofunction and significant reduction of Gsα and Giα may be found in the mononuclear leukocytes of depressed patients. 

However, some authors have suggested that Gqα, Giα, and Gβ levels were increased in the platelets of patients with depression [35] whereas no changes were reported in the granulocytes [36] or in mononuclear leukocytes [37] of the same subjects. Dwivedi *et al*. [12] found a significant decrease in RNA messenger and Gi2α levels associated with a relevant increase in levels of Gsα in the prefrontal cortex (PFC) of 43 postmortem brain suicide subjects compared with 38 nonpsychiatric control subjects. Similarly, Pacheco *et al*. [38] have reported a significant increase of Gsα in Brodmann area 10 and a significant decrease of Gi2α in Brodmann areas 8 and 9 of suicide subjects diagnosed with depression. They also found that suicide brain was hyperfunctional regarding adenylyl cyclase-cAMP activity and its receptor PKA. 

Abnormalities in G proteins coupled to cAMP signaling have also been reported in patients with bipolar disorder [34,39,40,41,42,43,44] and may potentially lead to dysfunctions of the downstream components of the cAMP dependent phosphorylation such as PKA and Rap1.

## 4. PKA, Depression and Suicidal Behaviour

PKA is a holoenzyme formed by regulatory and catalytic subunits; it is inactive in the absence of cAMP. When PKA regulatory subunits bind to cAMP, it causes changes in its conformational state, causing a dissociation of the regulatory subunitsfrom the catalytic (C) subunits. The catalytic subunit of PKA then binds to and phosphorylates biological targets. PKA consists of two domains, a smaller one with several β sheet structures and a larger one with several α helices. The binding sites for the PKA substrate and ATP are located in the catalytic cleft between the domains. When ATP and the substrate bind, the two lobes rotate so that the terminal phosphate group of the ATP and the target amino acid of the substrate move into the correct positions for the catalytic reaction to take place. PKA is controlled by cAMP: in the absence of cAMP, the kinase is a tetramer of two regulatory and two catalytic subunits (R2C2), with the regulatory subunits blocking the catalytic center of the catalytic subunits. The binding of cAMP to the regulatory subunit leads to the dissociation of active RC dimers. The catalytic subunit itself can be regulated by phosphorylation. Down-regulation of protein kinase A occurs by a feedback mechanism: one of the substrates that is activated by the kinase is a phosphodiestrase, which converts cAMP to AMP, thus reducing the amount of cAMP that can activate protein kinase A. In the nucleus, PKA modulates the activity of various cAMP-responsive - factors (e.g. CREB), which regulate the transcription of important genes like the gene encoding for the brain-derived neurotrophic factor, which has been demonstrated to be involved both in depression and suicide. Also, PKA may phosphorylate many substrates involved in neurotransmitter release, receptor desensitization, cell growth, differentiation, cell survival and synaptic plasticity [30,31,45,46]. Different PKA subtypes have different affinity for cAMP; three distinct catalytic subunits (Cα, Cβ, and Cγ) have been identified, but the exact functions of these different C subunits is unclear. The existence of different C subunits, no doubt, confers substantial diversity to the PKA mechanism of action at the intracellular level. Studies conducted to analyze tissue distribution of C subunits suggested that the subunit Cβ is particularly expressed in the brain [47], whereas Cα is widely expressed and Cγ is present only in the testis. 

Several studies heve investigated the role of the various PKA isoforms both in depression and suicidal behaviour. Dwivedi *et al*. [12] found a decreased activation of the regulatory subunits of PKA in the PFC of suicidal subjects compared to non psychiatric healthy control subjects both in presence and absence of cAMP. Similarly to Odagaki *et al*. [48], they also found a significant decrease in the mRNA and protein expression of selective regulatory RIIβ and catalytic Cβ subunits in the PFC of suicide subjects [13]. Pandey *et al*. [49] in a postmortem brain study of teenage suicidal subjects reported that mRNA expression levels of RIα and RIβ (not RIIβ or Cβ) were decreased in the PFC, but no changes were found when subjects were divided into those with a history of mental disorders and those with no such history. 

Dwivedi *et al*. [15] also found reduced levels of catalytic and regulatory PKA subunits. PKA activity has been reported to be significantly decreased in patients with affective disorders [20,21,22,23]. On the other hand, Perez *et al*. [50] have reported that the levels of the catalytic subunits of PKA were significantly higher in depressed and manic 52 euthymic patients, as compared to 62 healthy controls. 

Additionally, PKA plays a critical role in the regulation of motor and emotive behaviour, memory, stress, and also in the action of some psychotropic medications. RIIβ-mutant mice show important deficit in motor behaviour [51] and an increased ethanol consumption [52] whereas Cβ-mutant mice exhibit impaired hippocampal plasticity [53]. Targeted disruption of the RIβ subunit gene results in mice that exhibit defects in LTD, a deficit in synaptic plasticity, and specifically selective defects in mossy fiber long-term potentiation [51]. 

Shelton *et al*. [54] also recently anlyzed the expression of regulatory and catalytic PKA activity in post-mortem brain tissue of 20 melancholic, atypical, and non-classified depressed subjects compared to 20 age- and sex-matched controls. They showed a significantly lower expression of regulatory and catalytic PKA activity in the melancholic but not in the atypical subgroup. The melancholic subjects also had lower PKA RIα and Cβ expression in the frontal cortex, whereas those who died by suicide showed lower PKA RIα and Cα expression. The non-classified subgroup showed intermediary characteristics between atypical and melancholic subgroups.

Postmortem brain tissues analysis also showed that basal and cyclic AMP-stimulated PKA activity was reduced in 20 depressed subjects compared with 20 matched controls, while PKC activity was not different between groups [55]. McEwen [56] had previously reported similar findings describing the effects of glucocorticoids in terms of altered PKA activity in inducing neuronal hippocampal damage and death in severe psychosocial stress conditions.

Additionally, Manier *et al*. [57] have found a reduced activation of PKA in human fibroblasts of subjects with major depression and similarly, Shelton *et al*. [20] reported significantly less activity of beta-adrenoceptor-linked PKA in skin fibroblast samples of 12 depressed patients compared to 10 nondepressed volunteers. 

These data were confirmed by the same authors [21], who reported a reduced PKA activity in fibroblasts of 35 depressed melancholic subjects compared to 21 normal volunteers and Akin *et al*. [23], who later suggested lower levels of PKA RII alpha, C alpha, and C beta subunit isoform proteins associated with reduced CREB-P in skin fibroblasts of 12 depressed melancholic patients compared to 12 non-melancholic depressives and 12 normal controls.

PKA presumably plays an important role in the human ability to cope with stress and the acquisition of escape/avoidance responses [58,59,60,61]: aspects that are crucially involved in suicidal risk. However, whether a specific reduction of PKA subunits may reflect a clinical pathological phenotype is still largely unclear. Further longitudinal studies are needed to delineate this controversial point.

## 5. Rap-1, Epac-1 and Epac-2 in Depression and Suicide

Brains of depressed suicide victims show altered cAMP signaling and a decreased activation and expression of Rap-1, particularly in the prefrontal cortex (PFC) and hippocampus [15]. Rap1, a substrate of PKA, may be found in different tissues, including the brain and may be involved in important cellular events, such as cell survival, proliferation, cell adhesion, differentiation, and synaptic plasticity [62,63,64,65]. 

Rap-1 may also activate the extracellular regulated kinase (ERK) pathway [66], which is crucial for many cellular functions, such as neuroprotection, proliferation, cell survival and synaptic plasticity [67,68]. Since 1976, it’s well known that nerve growth factor treatment induces differentiation of cells into a sympathetic, neuron-like phenotype [69]. This effect appeared to be associated with a relevant activation of ERKs involving the PKA-dependent activation of the Ras-related small G protein Rap1 [70] and the activation of the isoform B-Raf [71,72].

Dwivedi and colleagues [14] have found a significantly decreased activation of ERK in PFC and hippocampus, but not in cerebellum of depressed suicide victims, hypothesizing that this reduction could be due to altered PKA levels or mediated by an abnormal ERK pathway [66]. In specific brain cells expressing B-Raf, PKA may stimulate the ERK pathway through the activation of Src-dependent kinases [73]. Ca^2+^-mediated PKA-dependent activation of Rap1/B-Raf/ERK signaling in hippocampal region is also, a crucial effector of neuronal functions [74]. Interestingly, Rap1 may link the PKA activity and ERK signaling via B-Raf and this link would explain how intracellular signaling systems comunicate with each other. Morozov *et al*. [75] demonstrated that mice with a dominant mutant of Rap-1 had both a reduced ERK levels in hippocampus, and a subsequent deficient learning and memory storage. Significantly decreased activation of ERK-1 and ERK-2 in the PFC and hippocampus of suicide subjects were found by Dwivedi *et al*. [76] in 11 depressed suicide subjects compared to 11 non-psychiatric control subjects. 

Rap-1 is activated by the GEF “Epac” containing a cAMP-binding site. There are two types of Epac: Epac-1, which is widely expressed in humans, and Epac-2, which is expressed in all brain regions and adrenal glands [77]. It has been reported that the level of Epac-1 is not changed whereas level of Epac-2 is significantly increased in PFC and hippocampus of depressed suicide victims [15]. However, many previous studies have reported higher cAMP dependent phosphorylation of Rap1 and increased levels (approximately of 20%) of Rap1 using immunoreactive techniques in platelets of untreated euthymic patients with bipolar disorder than in healthy individuals [78,79]. Perez *et al.* [50] have found that level of Rap1 is significantly higher in euthymic, depressed, and manic patients with bipolar disorder than in healthy subjects.

Perez *et al*. [80] also showed recently that among 29 untreated euthymic male bipolar outpatients the cAMP-dependent phosphorylation and the levels of Rap1 are significantly higher (of about 20%) as compared to healthy subjects suggesting that the abnormalities in the phosphorylation of Rap1 may reflect the increase in its own levels. These findings, taken together, show that the exact meaning of Rap1 abnormalities in depression and suicide remain still quite unclear.

Figure 2 provides informations about the role of Rap1 in in the transduction of intracellular signals. Rap1 is, a small GTP-binding protein, may be activated by the receptors coupled to G proteins-adenylate cyclase (cAMP) system [77,81,82]. through the phosphorylation by PKA at its C-terminal Ser-180 residue in a lot of cell types [62,83,84] above all neurons and glia cells [85,86,87] or by guanine nucleotide exchange factors directly activated by cAMP (cAMP-GEF) and also by Ca2+ and neurotrophic factors through a specific guanine nucleotide exchange factors (GEF). Additionally, a protein called Epac (exchange protein directly activated by cAMP), a guanine nucleotide- exchange factor activated by cAMP [88], may activate directly Rap-1. Epac has an autoinhibitory cAMP binding domain that facilitates the activation of Rap-1 by cAMP, independently of PKA [77,81,89]. cAMP is involved in a lot of neuro-synaptic mechanisms: recently it has been demonstrated that cAMP facilitates synaptic potentiation [90]. When activated, it bounds guanine nucleotide triphosphate (GTP), whereas if inactivated it is linked to guanine nucleotide diphosphate (GDP). Rap1 activates Raf, a serine-threonine kinase that may couple to neurotrophic factors, being crucial in the crosstalk between different signal transduction pathways. It‘s involved in a myriad of functions, such as cell proliferation and survival, cell adhesion, differentiation and synaptic plasticity [63,64,91].

## 6. Calcium, cADPR, Long-term Depression and Synaptic Plasticity

The intracellular messenger calcium (Ca^++^) is another crucial effector for synaptic transmission. Several metabolites of nicotinamide adenine dinucleotide (NAD^+^) such as the cyclic adenine dinucleotide phosphate ribose (cADPR) may participate as secondary messengers in the complex cell calcium signalling process [92]. cADPR modulates calcium movements from ryanodine-sensitive endoplasmic reticulum stores [93] and the activity of different plasma membrane calcium channels [94]. cADPR has been found in higher concentrations both in neurons [95,96,97,98] and in astrocytes [99], where it was demonstrated to be involved in neuronal excitability, synaptic transmission and neuronal plasticity [92]. Many investigators [100,101,102,103,104,105] suggest that cADPR may be produced after the activation of some metabotropic receptors and that it was involved in neurotransmitter release and long term synaptic depression [106,107,108]. 

**Figure 3:**
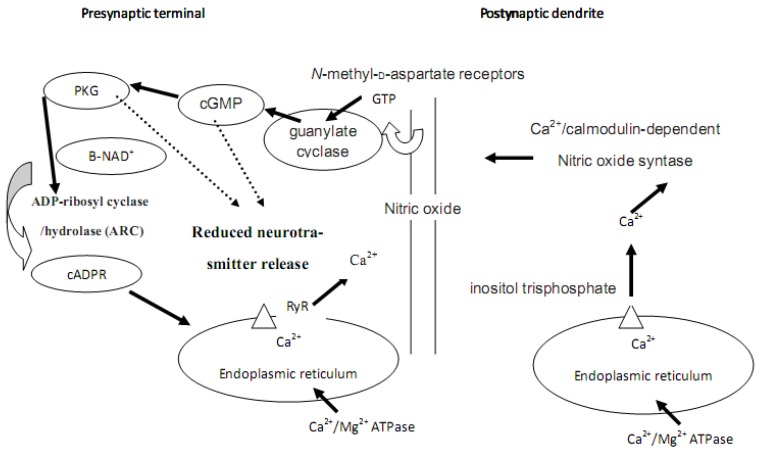
Calcium, cADPR, long term depression and synaptic plasticity. Adapted from [107].

cADPR has been reported to be crucial for the mammalian long term synaptic plasticity [96] and it could be the intracellular messenger linking cGMP to release from calcium stores, particulalry in the hippocampus, where high concentrations of cGMP may promote cADPR production. Presumably, Ca^2+^/calmodulin-dependent nitric oxide synthase produces the retrograde messenger nitric oxide (NO) diffusing to the presynaptic terminal activating guanylate cyclase to generate cGMP which activates PKG. PKG activates ADP-ribosyl cyclase/hydrolase producing cADPR, facilitating the release of calcium from intracellular stores (for more details see Figure 3) and induces long-term depression (LTD). cADPR may presumably be involved in processes like synaptic connections, synaptogenesis, and synaptic shaping during development.

Synaptic plasticity may be regulated through two bidirectional kinases: cGMP and cADPR (Figure 3), both necessary for LTD whereas cAMP and PKA promote LTP (Figure 2). The activation of PKG and the inhibition of PKA induce the production of cADPR and LTD [107]. However, these two distinct intracellular cascades act in opposite directions in the same synapse [107], suggesting the existence of independent mechanisms responsible for altered presynaptic transmitter release and postsynaptic sensitivity, both crucial for the expression of LTD. Similalry, both *N*-methyl-D-aspartate and metabotropic glutamate receptors stimulated by different stimuli were found to induce dependent forms of LTD [109], suggesting that different presynaptic activity patterns may activate one form in preference to another. 

It’s possible to assume that cADPR-Ca^++^ cascade presumably activates both presynaptic Ca^++^/calmodulin-dependent protein kinase II (CaMKII) and protein kinases A and C [107,110] (widely known as regulators of neuronal plasticity [111,112]. 

Neuronal calcium levels, no doubt, may regulate cell survival and synaptic plasticity through stimulation of intracellular kinase signaling pathways involving in the induction of gene expression and long term potentiation. Relevant effectors like Rap1 and B-Raf are highly expressed in the central nervous system and may regulate a number of activity-dependent neuronal functions. However, how all these different mechanisms through neuronal calcium variability act leading to synaptic plasticity, are still unclear. 

## 7. The Pivotal Role of PKA in Synaptic Plasticity and Cell Survival

Biologically, some glycone or aglycone parts of the glycosides may act on specific intracellular enzymes such as protein kinases. Several glycosides, which play important biological roles in the brain presumably act through the regulation of targeted plasticity-related proteins such as PKA. Although it’s possible to speculate that abnormalities in PKA are critical in the pathophysiology of depression and suicidal behaviour, how PKA exactly contributes in terms of pathogenesis is still matter of debate. Rap1 and the transcription factor CREB seem to be crucial substrates, through which PKA alterations are linked to suicidal behaviour. Unfortunately, it remains quite difficult to elucidate the molecular mechanisms underlying the abnormalities of both Rap1 and PKA.

Since 1997, Kandel *et al*. [113] have found in aplysia that LTF is synapse-specific and requires the nucleus presumably implying local translation of mRNAs and protein synthesis. In particular, stimuli inducing LTF, increase both ERK activity and ERK nuclear localization. In mammalians, LTP-inducing stimuli might specifically increase the expression of ERK2 and B-Raf in the hippocampus. ERK is essential for hippocampal LTP induction [114] reflecting that ERKs are required for cAMP-responsive element binding protein (CREB)-mediated gene transcription [115]. LTP is, however, a multifaceted phenomenon; so, it is unclear whether they reflect changes in pre- or postsynaptic functions, and also the mechanisms that regulate them may vary in function in the cerebral region which was examined. ERKs targets may be both nuclear (e.g. CREB-dependent transcription [115] and cytoplasmic (e.g. ApCAM [116]). ERKs include both modulation of ion-channel function [117] and direct activation of the cell protein synthesis [118,119]. Additionally, ERKs may directly phosphorylate synapsin I [120] and this reflects the mechanism by which neurotrophins exert rapid effects on neurotransmission. Ca^++^ release from presynaptic and postsynaptic intracellular stores is crucial for long-term depression (LTD) and long-term potentiation (LTP) of synaptic signaling [107,112,121,122]. Long-term activity-dependent changes in synaptic efficacy represent a relevant method of memory storage and synaptic transmission/plasticity are strictly dependent on the intracellular levels of Ca^++^. Interestingly, LTP represent the main molecular mechanism underlying learning and memory; so, a role for ERKs, which is directly involved in hippocampal LTP, has been clearly identified also for learning and memory. 

Taken together, these studies provide strong evidence that ERKs are important components of activity-dependent signaling cascades within neurons and that modulation of their activity may be required for both synaptic plasticity, learning and memory. Finally, the ability of PKA and Rap1 to couple to ERK activation may have significant implications for neuronal signaling [123]. PKA stimulation of ERK activity may also regulate both neuronal survival and synaptic plasticity [113,124]. 

But, may we consider PKA and ERK disturbances as state related abnormalities or disease dependent alterations? Perez *et al*. [50] suggested that altered levels of Rap1 observed in the whole sample of patients with bipolar disorder (112 drug-free patients with bipolar disorder (52 euthymic, 29 depressed and 31 manic) could be considered a state-independent biochemical abnormality, whereas altered levels of PKA observed only in their depressed and manic patients (unvaried levels in euthymic patients) could be considered a state related abnormality. Perez *et al*. [125], assessing the levels of PKA and Rap1 in platelets of 45 drug-free unipolar patients (13 euthymic and 32 with major unipolar depression depressed), found that the regulatory subunit type II of protein kinase A and Rap1 were significantly lower in untreated depressed patients compared with untreated euthymic patients and healthy subjects. Perez *et al*. [50,80,126] hypothesized that PKA is differentially altered in psychotic and nonpsychotic patients with major depressed unipolar and bipolar disorder. Both unipolar and bipolar (37 drug-free patients) with psychotic depression have been reported to have significantly lower levels of platelet regulatory type I and higher levels of catalytic PKA subunits than 29 healthy controls, whereas the levels of regulatory type II were high only in psychotic unipolar patients [125].

Also, some authors have reported that alterations in some components of cAMP signaling in affective patients may be treatment-related rather than disease related [127]. They investigated CREB concentrations in 60 postmortem brain tissues of untreated patients and those treated with antidepressants (15 with major depression, 15 with bipolar disorder, 15 with schizophrenia, and 15 with nonpsychiatric controls). Only in in the major depression group, higher temporal but not occipital cortex CREB concentrations were found in patients treated with antidepressants than in untreated patients who, in turn had lower CREB concentrations than controls whereas no differences were reported between patients with bipolar disorder or schizophrenia and controls or between treated and untreated patients with bipolar disorder and schizophrenia.

However, whether the presented abnormalities are primary disease-related changes or possibly reflecting adaptive responses secondary to other dysfunctions in cell signaling remained still unclear. Further additional studies are needed to clarify the exact relationship between the levels of Rap1 and catalytic subunits in patients with affective disorders and suicidal risk.

## 8. CREB and BDNF in Synaptic Plasticity and Cell Survival

The correct regulation of target gene expression is a crucial step for brain development, homeostasis, and adaptation to the environment stressors. CREB is an important transcription factor which is activated by the phosphorilation at serine 133 by PKA, which may then activate or repress the transcription of specific target genes [30]. CREB is involved in neuronal functioning, excitation of nerve cells, central nervous system development, long-term synaptic plasticity [70], learning, memory, and cell survival [121,128,129,130,131].

It has been found that both the phosphorylation/expression of CREB and the PKA activity are reduced in postmortem brain of patients with depression [132] and of depressed suicide victims, specifically in the PFC, hippocampus [19,133] and amygdala [41]. Additionally, as mentioned earlier, the expression of CREB may be activated or upregulated by antidepressant drugs [127,134]. Zubenko *et al*. [135], while conducting a linkage analysis of six polymorphic markers in a specific region of chromosome 2q33–35, found a linkage between depression and a specific region of the chromosome 2q33–34 containing the CREB1 gene. Nagakawa *et al*. [136] aimed to determine the influence of cAMP cascade on neurogenesis found that rolipram, an inhibitor of cAMP breakdown, may activate the cAMP cascade (CREB phosphorylation) and increase the proliferation of new granule cells in the dentate gyrus cells of adult mouse expressing neuronal-specific markers. Several studies confirmed that the cell proliferation is reduced in conditional transgenic mice that express a dominant negative mutant of CREB in hippocampus [137,138]. Gur *et al*. [139] have found that the antidepressant phenotype is associated with increased cell proliferation and neurogenesis in CREB-deficient mice. 

However, not all studies in literature confirm the role of BDNF and CREB in depression and suicidal behaviour. Groves [140] critically reviewing all the clinical and preclinical studies regarding the BDNF hypothesis of depression stated that a loss of BDNF is directly involved in the pathophysiology of depression.

No change in cAMP binding to regulatory subunits of PKA were found in postmortem brains of depressed suicide subjects [141], while an increased expression of total and phosphorylated active CREB was reported in the PFC of depreesed suicide subjects [48]. 

But PKA/CREB may also activate the transcription of the BDNF participating in neurite outgrowth, phenotypic maturation, morphological plasticity [142], nerve regeneration, structural integrity and synaptic activity [143,144,145]. It was demonstrated that CREB activation is crucial for the upstream regulation of BDNF after chronic antidepressant therapy [146]. 

Several lines of evidence suggest that BDNF may play a crucial role in the pathophysiology of suicide. Dwivedi *et al*. [133] found in postmortem brain tissues of suicide victims that the mRNA and protein levels of BDNF were significantly and homogeneously reduced in both the PFC and hippocampus of suicide subjects irrespective of psychiatric diagnosis, suggesting that there is a presumably defect in the transcription of the BDNF gene. Karege *et al*. [147] reported that the level of BDNF, in suicide victims but not in those subjects who were receiving antidepressant drugs, was significantly lower in the PFC and hippocampus but not in the entorhinal cortex. 

Considering that similar levels of plasma BDNF and brain BDNF levels were found [148], Kim *et al*. [149] reported that plasma BDNF levels was significantly lower in 32 major depressed patients who had recently attempted suicide compared with 32 non-suicidal depressed patients and 30 normal controls. The authors suggested that although BDNF levels were not able to distinguish between lethal and non-lethal suicide attempts, a reduction of plasma BDNF level may be considered as a biological marker of suicidal depression. 

Sarchiapone *et al*. [150], investigating 97 depressed patients carrying the BDNF Val66Met polymorphism variant who had attempted suicide, found that they showed a significantly increased risk of suicidal behaviour, particularly if they reported higher levels of childhood emotional, physical and sexual abuse compared to 73 who had not attempted suicide.

Kohli *et al*. [151], trying to determine whether the BDNF gene or its high-affinity receptor gene, receptor tyrosine kinase 2 (NTRK2), confer risk for suicide attempt and major depression, found that in 394 depressed patients, of whom 113 had suicide attempts, independent single-nucleotide polymorphisms within NTRK2 were associated with suicide attempts among depressed patients. Patients carrying risk genotypes in all three markers were at higher suicidal risk (more than four-fold) compared with those without any of the three risk genotypes.

However, other studies suggested that plasma BDNF level was lower in both the attempted suicide group and the depressed group than the control group [152] or that serum BDNF level was lower in 20 major depressed non-suicidal subjects and 20 depressed subjects who have recently attempted suicide compared to healthy controls [153]. Dawood *et al*. [154] found that venoarterial BDNF concentration gradient was significantly decreased in patients at medium to high suicide risk in depressed drug-free patients. Finally, although a large body of evidence suggested that dysfunctional neurotrophic signaling might be involved in the pathophysiology of complex neurobiological disorders, further additional studies are required to exactly clarify the role of neurotrophins in determining depression and suicidal behaviour.

## 9. Conclusions: Is There Evidence of A Central Role For Glycosides And Glycoside-Linked Signal Transduction Proteins In Depression and Suicide?

Some glycosides play different biological roles presumably regulating the expression of some glycoside-linked signal transduction and plasticity-related proteins such as PKA and G proteins, able to activate several intracellular signaling pathways. Abnormalities in these glycosides function may reflect abnormal plasticity of neuronal pathways presumably implicated in the pathogenesis of mood disorders/suicidal behaviour [155]. Both affective disorders and suicide may emerge from the brain inability to give a adequate adaptive response to environmental stimuli derived from abnormal neuronal plasticity [156,157,158]. 

Synaptic plasticity may be altered in different forms: reductions in cell number, cell density, cell body size, neuronal and glial density primarily in frontal cortex or hippocampal brain areas, parahippocampal cortex and cortical laminar thickness [159,160,161,162,163,164]. Additionally, abnormalities in synaptic circuitry [165], synaptic connectivity [166], number and shape of dendritic spines [167], synapse formation [56], neuronal atrophy [168], and spatial cognition deficits [169] have been found both in depression and under stress conditions. Several recent pieces of evidence [6,14,15,16,49,133,170,171,172,173,16,49,133,170] have suggested that these abnormalities may be the result of altered molecular mechanisms involving in the structural/neural plasticity and cellular resilience. 

**Figure 4:**
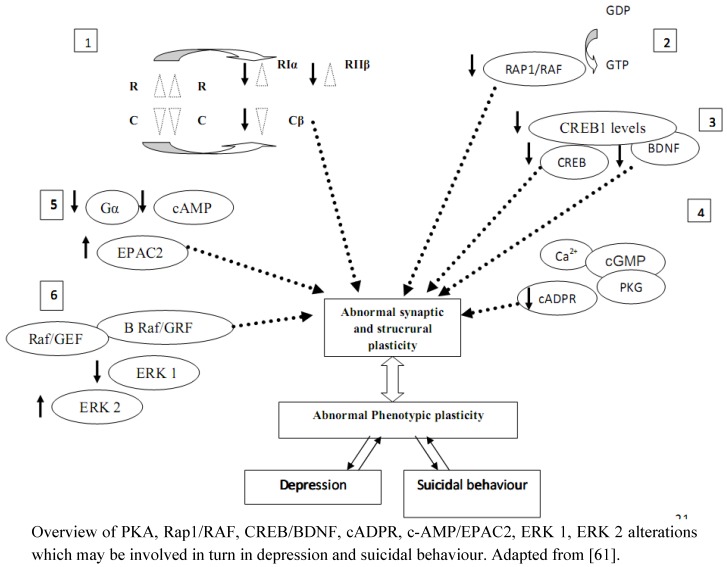
Alterations of intracellular substrates, depression and synaptic plasticity.

Abnormalities in specific intracellular signals mediated by effectors like PKA, CREB and BDNF seem to have a crucial role in regulating neural plasticity, determining behavioural responses and adaptations to environmental stressful events as well as cell survival, synaptic plasticity, and activation or repression of gene expression in the patophysiology of affective disorders and suicidal behaviour [30,31]. However, whether these changes in specific biological molecular targets are specific to suicide or generally related to mental disorders is still matter of debate (Figure 4).

A new neurobiological model including some biological abnormalities in PKA/Rap1/CREB/BDNF as relevant biological risk markers is gradually advancing in order to clarify the various aspects of the complex patophisiology of mental illnesses and eventually develop site-specific therapeutic interventions. Further additional studies are needed to deeply investigate the multifaced aspects underlying affective disorders and suicidal behaviour.
